# Prognostic value of the hemolysis index in patients with significant hemolysis

**DOI:** 10.1186/cc14666

**Published:** 2015-09-28

**Authors:** Diego O Cortés, Arthur Cezar M Xavier, Bruno R Almeida, Érica C Vieira, Jacques Creteur, Jean-Louis Vincent, Joao Claudio Lyra, Sylmara Zandona

**Affiliations:** 1 Department of Intensive Care, Hôpital Erasme, Anderlecht, Brussels, Belgium

## Introduction

Hemolysis is a frequent complication of different extracorporeal circulation and membrane oxygenation (ECMO) support systems. Usually it is assessed by measuring the levels of haptoglobin or the concentrations of free hemoglobin in the plasma, but automated biochemical laboratory analyzers now detect the hemolysis index (HI) of all blood samples as a measure of sample quality. We studied whether this simple index could detect populations at high risk of active hemolysis and whether it is correlated with outcome.

## Methods

We evaluated all admissions to our department of intensive care during 2013 and collected relevant demographic and organ dysfunction data during the first 24 hours as required for the SOFA score (not the neurological component). We also collected data on whether or not the patients needed renal replacement therapy during the ICU stay. Patients were classified into three groups: those who needed ECMO support during the ICU stay, those who were admitted after cardiac surgery and had cardiopulmonary bypass (CPB), and other patients. We compared the initial and median (throughout the ICU stay) HI values in the different groups and the survivors with the nonsurvivors. We used SPSS 22.0 (IBM, USA) for all analyses and a *p *value < 0.05 was considered as significant.

## Results

We studied 2021 patients with the characteristics presented in Table 1. Patients treated with ECMO and cardiac surgery patients had higher initial and median HI values than the other patients. The nonsurvivors in the ECMO group had higher median HI values than survivors (4 (2-21) vs. 2 (1-3), p < 0.01). There were no differences in the initial or median HI values between patients treated or not with renal replacement therapy.

**Table 1:** Characteristics and hemolysis index in different groups.

	ECMO (*n *= 56)	CBP (*n *= 246)	Others (*n *= 1719)	*p *value
Age (years)	53 (38-61)	68 (59-78)	59 (45-70)	<0.01
Emergency surgery, *n *(%)	4 (7)	10 (4)	177 (10)	<0.01
Lowest mean arterial pressure in first 24 hours (mmHg)	64 (60-69)	65 (61-69)	71 (64-82)	<0.01
Norepinephrine use in first 24 hours, *n *(%)	49 (88)	143 (58)	363 (21)	<0.01
Renal replacement therapy during ICU stay, *n *(%)	22 (39)	13 (5)	109 (6)	<0.01
Transfusion during first 48 hours, *n *(%)	41 (73)	76 (31)	223 (13)	<0.01
Renal failure at admission, *n *(%)	35 (63)	51 (21)	440 (26)	<0.01
SOFA score at admission	9 (7-11)	6 (4-7)	3 (1-5)	<0.01
Mortality, *n *(%)	26 (46)	15 (6)	186 (11)	<0.01
Initial hemolysis index	7 (2-18)	11 (4-21)	2 (0-4)	<0.01
Median hemolysis index	2 (1-4)	3 (1-5)	2 (0-3)	<0.01

## Conclusion

Patients undergoing CBP for cardiac surgery and those treated with ECMO support had higher HI values measured automatically in contemporary analyzers. The HI had prognostic value in patients treated with ECMO.

